# Plant tissue characteristics of *Miscanthus x giganteus*

**DOI:** 10.1038/s41597-022-01424-0

**Published:** 2022-06-15

**Authors:** Oliva Pisani, Dan Liebert, Timothy C. Strickland, Alisa W. Coffin

**Affiliations:** USDA-ARS, Southeast Watershed Research Laboratory, Tifton, GA USA

**Keywords:** Environmental chemistry, Agroecology

## Abstract

As part of a study identifying relationships between environmental variables and insect distributions within a bioenergy crop, giant miscanthus (*Miscanthus x giganteus*) samples were collected in October 2016 at 33 locations within a field in southeast Georgia, USA. At each location, one plant sample was collected every 3 to 4 m along a 15 m transect, resulting in 5 replicates per sampling location. The plant samples were separated into leaves and stems, dried, and ground. The chemical composition of the ground material was assessed by measuring total carbon and nitrogen, total macro- and micronutrients (aluminum, arsenic, boron, calcium, cadmium, cobalt, chromium, copper, iron, potassium, magnesium, manganese, molybdenum, sodium, nickel, phosphorus, lead, sulfur, selenium, silicon, titanium, vanadium, and zinc) using Inductively Coupled Plasma with Optical Emission Spectroscopy (ICP-OES), and optical characteristics of the water extractable organic matter (WEOM) using UV-Visible and Fluorescence Excitation Emission Matrix (EEM) spectroscopy. This dataset will be useful to identify relationships between the chemical composition of giant miscanthus tissues and pest distributions within a bioenergy crop field.

## Abstract (summary)

**Table:** 

Measurement(s)	plant tissue chemical composition
Technology Type(s)	inductively coupled plasma with optical emisison spectroscopy • UV-Visible and fluorescence spectroscopy
Sample Characteristic - Organism	Miscanthus x giganteus
Sample Characteristic - Environment	bioenergy crop field
Sample Characteristic - Location	United States

## Background & Summary

*Miscanthus x giganteus* (or giant miscanthus) is a perennial grass used increasingly as a bioenergy feedstock^1^. In the United States, the use of giant miscanthus has increased as producers seek productive biofeedstock alternatives to corn (*Zea mays*)^2^ that are more suitable for planting in marginal lands^3^. However, as a relatively new crop, we have only preliminary knowledge about insect pests of giant miscanthus, including the drivers of infestations and plant responses. Therefore, a study was undertaken in Georgia, USA, in 2015 and 2016 to ascertain more about the dynamics among insect pests, giant miscanthus plants, and broader field characteristics^4^. This work showed that wind speed, plant health, and soils are important drivers of phloem-feeding insect numbers but that these can vary over space and time. The dataset described here builds on an earlier dataset^5^ to provide detailed chemical characteristics of giant miscanthus plant tissues associated with the studies described by Coffin *et al*.^3,4^. These data will be useful to researchers focused on improving knowledge about the relationships among arthropod communities in giant miscanthus described earlier, and giant miscanthus plant tissue characteristics described here.

**Fig. 1:**
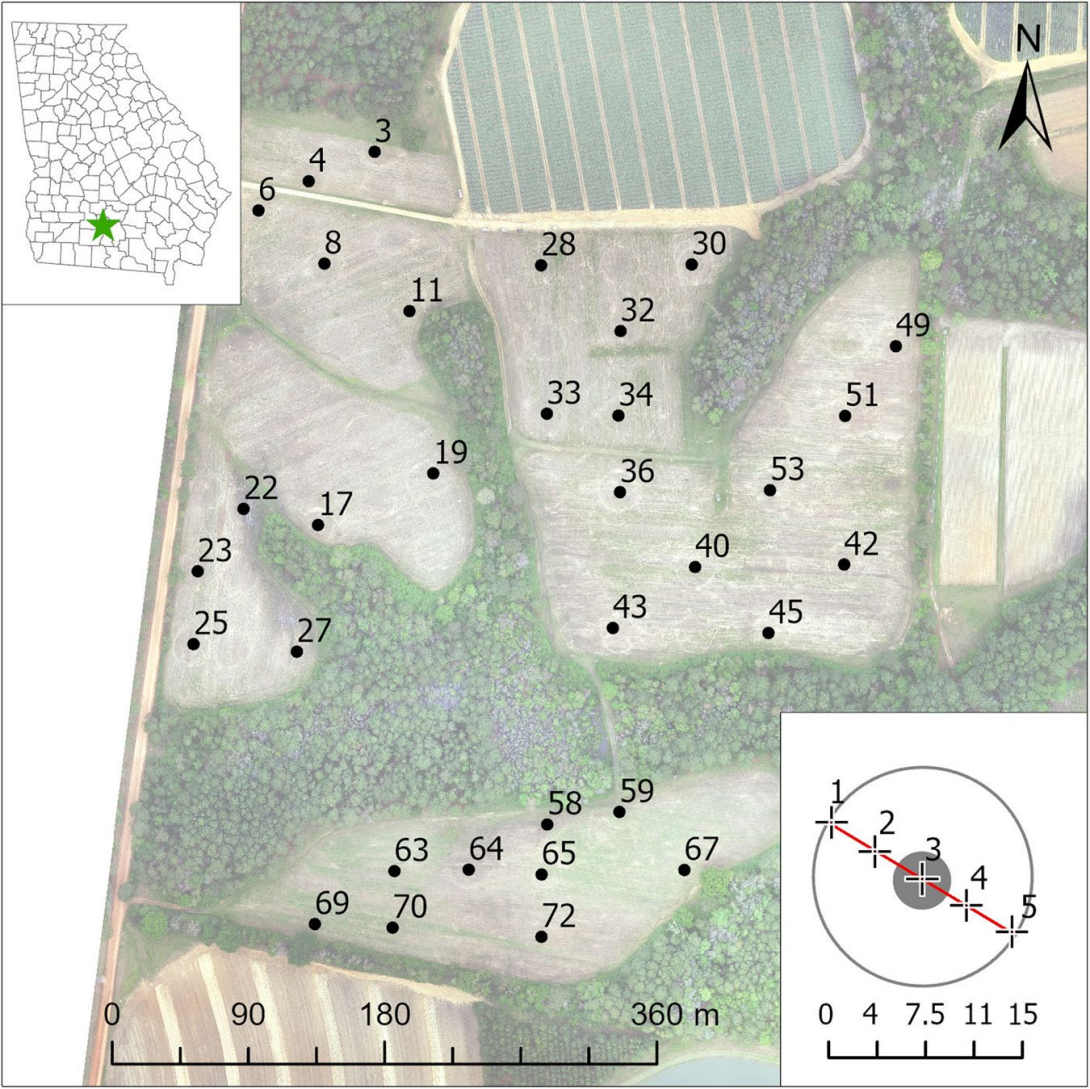
Map of *Miscanthus x giganteus* data collection locations. Center panel – map of plant tissue sampling locations^4^. Upper left inset panel – general study location in TyTy, GA, denoted by a green star. Lower right inset panel – diagram of typical 15 m transects (red line centered, in gray, and extending across each circular plot), showing locations of sample collection points (black crosses) every ~3.75 m along the transect.

During a study conducted in 2016, giant miscanthus leaf and stem tissue samples were collected at 33 locations within a field, co-located with contemporaneous arthropod collection efforts (Fig. 1). Field management practices were previously described by Coffin *et al*.^3,4^. After completion of the arthropod collection campaign^4^, circular plots (r ≈ 8 m) centered at each sampling location were marked, and the crop was harvested from the surrounding areas. Aboveground plant tissue collection then occurred on 12 October 2016 along a 15 m transect centered on the undisturbed sampling locations, where one sample was collected every ~3.75 m, providing 5 sub-samples, or “reps” per circular plot.

## Methods

### Geospatial data

Sampling locations were established, flagged, and recorded in June 2016, using a Trimble Geo7X global navigation satellite system (GNSS) receiver using the Trimble® VRS Now real-time kinematic (RTK) correction. Location accuracies were verified to within ±2 cm. Points were imported into a geodatabase using Esri ArcMap (Advanced license, Version 10.5) and projected using the Universal Transverse Mercator (UTM), Zone 17 North projection, with the 1983 North American datum (NAD83). Field investigators navigated to the flagged locations by visually locating them in the field or by using recreational grade GNSS receivers with the locations stored as waypoints.

### Plant tissue sampling and preparation

**Fig. 2:**
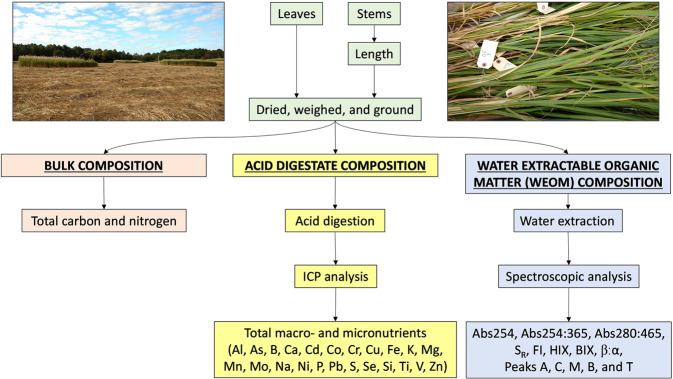
Images of field samples, and diagram of plant tissue processing. Center panel – flow chart outlining the procedures for plant tissue processing, the kinds of analyses performed, and the type of data generated. Upper left inset panel – ground level picture of *Miscanthus x giganteus* circular plots. Upper right inset panel – some plant samples on the day of collection.

*Miscanthus x giganteus* grows in clumps of bamboo-like canes. A single cane was cut at soil level from each of the five sample collection points in each circular plot, individually labelled, and brought to the lab for processing (Fig. 2). Each stem was measured from the cut at the base to the last leaf node, and the length was recorded. Green, fully expanded leaves were cut from each stem and leaves and stems from each plant were placed in separate paper bags and dried at 60 °C. The dry leaf and stem tissues were ground to pass a 1 mm screen (Wiley Mill Model 4, Thomas Scientific, Swedesboro, New Jersey, USA). Subsamples of the ground material were analyzed for total carbon (C) and nitrogen (N), acid-digested for the analysis of total macro- and micronutrients, and water-extracted for spectroscopic analysis and the characterization of the water extractable organic matter (WEOM) (Fig. 2).

### Total carbon and nitrogen

Dried and ground leaf and stem material (~4–6 mg) was analyzed for total C and N content by combustion (Vario EL III, Elementar Americas Inc., Mt. Laurel, New Jersey, USA). The instrument was calibrated using an aspartic acid standard (36.08% C ± 0.52% and 10.53% N ± 0.18%). Validation by inclusion of two aspartic acid samples as checks in each autosampler carousel (80 wells) resulted in a net positive bias of 1.44 and 1.68% for C and N, respectively. The mean C and N concentrations and standard deviations for the sample set are presented in Table 1.

**Table 1:** Giant miscanthus composition including leaf (L) and stem (S) dry weight, length, and carbon (C) and nitrogen (N) concentrations (*n* = 165). Values are reported as means ± standard deviations.

Leaf/Stem	Mean dry weight (g)	Mean length (cm)	Mean C concentration (% dry weight)	Mean N concentration (% dry weight)
L	2.99 ± 1.99	NA	44.66 ± 1.09	1.11 ± 0.30
S	9.39 ± 5.68	127.04 ± 51.46	45.08 ± 1.04	0.30 ± 0.10

### Macro- and micronutrients

Plant tissue samples were analyzed for a suite of macro- and micronutrients including aluminum (Al), arsenic (As), boron (B), calcium (Ca), cadmium (Cd), cobalt (Co), chromium (Cr), copper (Cu), iron (Fe), potassium (K), magnesium (Mg), manganese (Mn), molybdenum (Mo), sodium (Na), nickel (Ni), phosphorus (P), lead (Pb), sulfur (S), selenium (Se), silicon (Si), titanium (Ti), vanadium (V), and zinc (Zn) using Inductively Coupled Plasma with Optical Emission Spectroscopy (ICP-OES). Samples (0.5 g) were digested using 10 mL of trace metal grade nitric acid (HNO_3_) in a microwave digestion system (Mars 6, CEM, Matthews, North Carolina, USA). During the digestion procedure (CEM Mars 6 Plant Material Method), the oven temperature was increased from room temperature to 200 °C in 15 minutes and held at 200 °C for 10 minutes. The pressure limit of the digestion vessels was set to 800 psi although it was not monitored during individual runs. Sample digestates were transferred quantitatively to centrifuge tubes, diluted to 50 mL with 2% HNO_3_ (prepared with lab grade deionized water), and centrifuged at 2500 rpm for 10 min (Sorvall ST8 centrifuge, Thermo Fisher Scientific, San Jose, California, USA). The digestates were decanted into clean centrifuge tubes and analyzed using an iCAP 7400 ICP-OES Duo equipped with a Charge Injection Device detector (Thermo Fisher Scientific, San Jose, California, USA). An aliquot of digested sample was aspirated from the centrifuge tube using a CETAC ASX-520 autosampler (Teledyne CETAC Technologies, Omaha, Nebraska, USA) and passed through a concentric tube nebulizer. The resulting aerosol was then swept through the plasma using argon as the carrier gas with a flow rate of 0.5 L/min and a nebulizer gas flow rate of 0.7 L/min. Macro- and micronutrients were quantified by monitoring the emission wavelengths (Em λ) reported in Table 2.

**Table 2:** Macro- and micronutrients measured, and emission wavelengths (Em λ) used to quantify them in the miscanthus leaves (L) and stems (S), the total number and percentage detected (*n* = 150 for leaves and 162 for stems), the mean detected concentration ± standard deviation, and the mean method detection limit (MDL) ± standard deviation.

Element	Em λ (nm)	Leaf/Stem	# detected	% detected	Mean concentration (mg/kg)	Mean MDL (mg/kg)
Al	308.2	L	150	100	44.5 ± 24.3	7.48 ± 2.81
		S	98	60	9.30 ± 7.08	6.88 ± 0.903
As	193.7	L	0	0	0.00137 ± 0.0119	1.18 ± 0.443
		S	0	0	0.00768 ± 0.0598	1.08 ± 0.142
B	249.7	L	150	100	40.0 ± 40.3	0.935 ± 0.351
		S	161	99	6.83 ± 14.2	0.859 ± 0.113
Ca	317.9	L	150	100	6309 ± 2418	48.7 ± 18.3
		S	162	100	1588 ± 954	44.8 ± 5.88
Cd	226.5	L	0	0	0.00167 ± 0.00448	0.115 ± 0.0432
		S	0	0	0.0110 ± 0.0140	0.106 ± 0.0139
Co	230.7	L	13	9	0.0324 ± 0.0537	0.115 ± 0.0433
		S	10	6	0.0307 ± 0.0453	0.106 ± 0.0139
Cr	205.5	L	134	89	0.589 ± 0.329	0.232 ± 0.0871
		S	126	78	0.409 ± 0.276	0.213 ± 0.0280
Cu	224.7	L	148	99	4.23 ± 1.53	1.20 ± 0.0450
		S	135	83	1.73 ± 0.630	1.10 ± 0.145
Fe	238.2	L	150	100	76.6 ± 38.6	1.15 ± 0.433
		S	162	100	23.7 ± 11.0	1.06 ± 0.139
K	766.4	L	150	100	10778 ± 3129	88.5 ± 33.3
		S	162	100	8296 ± 2476	81.4 ± 10.7
Mg	279.0	L	150	100	1008 ± 361	7.22 ± 2.71
		S	162	100	671 ± 242	6.64 ± 0.871
Mn	257.6	L	150	100	142 ± 128	1.20 ± 0.450
		S	162	100	129 ± 123	1.10 ± 0.144
Mo	202.0	L	119	79	0.158 ± 0.0612	0.114 ± 0.0427
		S	10	6	0.0582 ± 0.0345	0.105 ± 0.0137
Na	589.5	L	110	73	22.1 ± 11.2	15.1 ± 5.66
		S	89	55	16.3 ± 7.90	13.8 ± 1.82
Ni	231.6	L	144	96	0.272 ± 0.171	0.115 ± 0.0432
		S	140	86	0.221 ± 0.184	0.106 ± 0.0139
P	178.2	L	150	100	1355 ± 349	0.917 ± 0.345
		S	162	100	852 ± 516	0.843 ± 0.111
Pb	220.3	L	150	100	1.15 ± 0.699	0.115 ± 0.0431
		S	162	100	0.561 ± 0.355	0.105 ± 0.0138
S	182.0	L	150	100	1026 ± 171	7.51 ± 2.82
		S	162	100	543 ± 256	6.90 ± 0.905
Se	196.0	L	73	49	1.18 ± 0.440	1.15 ± 0.432
		S	19	12	0.852 ± 0.228	1.06 ± 0.139
Si	251.6	L	150	100	1283 ± 473	41.7 ± 15.7
		S	162	100	569 ± 266	38.3 ± 5.03
Ti	334.9	L	150	100	0.766 ± 0.323	0.113 ± 0.0426
		S	134	83	0.185 ± 0.0985	0.104 ± 0.0137
V	292.4	L	18	12	0.143 ± 0.144	0.321 ± 0.120
		S	3	2	0.0973 ± 0.0984	0.295 ± 0.0387
Zn	213.8	L	146	97	19.3 ± 5.21	9.26 ± 3.48
		S	142	88	18.6 ± 15.1	8.52 ± 1.12

### Characterization of the water extractable organic matter (WEOM)

The WEOM of the giant miscanthus leaves and stems was isolated by extracting the plant material with deionized water at room temperature^6^. The water extractions were performed by mixing ~0.2 g of dry, ground leaves and stems with 100 mL of deionized water in 125 mL pre-washed brown Nalgene bottles. All brown Nalgene bottles used for these extractions were pre-washed by soaking them for 24 hours in a 10% hydrochloric acid solution followed by 24 hours in a 10% sodium hydroxide solution, and a thorough rinse with deionized water. The bottles containing the extraction solution were shaken on an orbital shaker at 180 rpm for 24 hours. The extract was vacuum filtered using 0.45 µm glass fibre filters (GF/F, Whatman) into pre-washed 60 mL brown Nalgene bottles. The filtered water extracts containing the WEOM were stored in the dark in a refrigerator (4 °C) until analysis by UV-Visible and fluorescence spectroscopy. Samples were visually inspected just prior to analysis to ensure no colloids or precipitates had formed during storage. Samples that had become visually cloudy were re-filtered.

On the day of analysis, the water extracts were removed from the refrigerator and allowed to warm up to room temperature. Chemical characteristics of the WEOM were assessed through the analysis of optical properties on an Aqualog spectrofluorometer (Horiba Scientific, New Jersey, USA) equipped with a 150 W continuous output Xenon arc lamp. Excitation-emission matrix (EEM) scans were acquired in a 1 cm quartz cuvette with excitation wavelengths (Ex λ) scanned using a double-grating monochrometer from 240 to 621 nm at 3 nm intervals. Emission wavelengths (Em λ) were scanned from 246 to 693 nm at 2 nm intervals and emission spectra were collected using a Charge Coupled Device (CCD) detector. All fluorescence spectra were acquired in sample over reference ratio mode to account for potential fluctuations and wavelength dependency of the excitation lamp output. Samples were corrected for the inner filter effect^7^ and each sample EEM underwent spectral subtraction with a deionized water blank to remove the effects due to Raman scattering. Rayleigh masking was applied to remove the signal intensities for both the first and second order Rayleigh lines. Instrument bias related to wavelength-dependent efficiencies of the specific instrument’s optical components (gratings, mirrors, etc.) was automatically corrected by the Aqualog software after each spectral acquisition. The fluorescence intensities were normalized to the area under the water Raman peak collected on each day of analysis and are expressed in Raman-normalized intensity units (RU). All sample EEM processing was performed with the Aqualog software (version 4.0.0.86).

The optical data obtained from the EEM scans were used to calculate several indices representative of WEOM chemical composition (Table 3) including the absorbance at 254 nm (Abs254), the ratio of the absorbance at 254 to 365 nm (Abs254:365), the ratio of the absorbance at 280 to 465 nm (Abs280:465), the spectral slope ratio (S_R_), the fluorescence index (FI), the humification index (HIX), the biological index (BIX), and the freshness index (β:α). The S_R_ was calculated as the ratio of two spectral slope regions of the absorbance spectra (275–295 and 350–400 nm)^8^. The FI was calculated as the ratio of the emission intensities at Em λ 470 and 520 nm, at an Ex λ of 370 nm^9^. The HIX was calculated by dividing the emission intensity in the 435–480 nm region by the sum of emission intensities in the 300–345 and 435–480 nm regions, at an Ex λ of 255 nm^10^. The BIX was calculated as the ratio of emission intensities at 380 and 430 nm, at an Ex λ of 310 nm^11^. The freshness index β:α was calculated as the emission intensity at 380 nm divided by the maximum emission intensity between 420 and 432 nm, at an Ex λ of 310 nm^12^. To further characterize the giant miscanthus WEOM, the fluorescence intensity at specific excitation-emission pairs was also identified. The fluorescence peaks identified here have previously been reported for surface water samples and water extracts^13^ and include peak A (Ex λ 260, Em λ 450), peak C (Ex λ 340, Em λ 440), peak M (Ex λ 300, Em λ 390), peak B (Ex λ 275, Em λ 310), and peak T (Ex λ 275, Em λ 340). A brief description of these optical indices is provided in Table 3.

**Table 3:** Description of the optical indices calculated from the excitation-emission matrix (EEM) fluorescence scans and used to analyze the WEOM composition of giant miscanthus leaves and stems.

Measurement	Calculation	Purpose
Abs254	Absorbance at 254 nm.	A greater value is associated with a greater aromatic content.
Abs254:365	Absorbance at 254 nm divided by the absorbance at 365 nm.	A low value is associated with low molecular weight material with a low degree of transformation.
Abs280:465	Absorbance at 280 nm divided by the absorbance at 465 nm.	A low value is associated with low molecular weight material with a low degree of transformation.
Spectral slope ratio (S_R_)^8^	Spectral slope from 275–295 nm divided by the spectral slope from 350–400 nm.	Negatively correlated to molecular weight.
Fluorescence Index^7,9^	The ratio of emission intensity at 470 and 520 nm, at an excitation of 370 nm.	Relative contribution of terrestrial and microbial sources to the organic matter pool.
Humification Index^10^	The area under the emission spectrum from 435–480 nm divided by the sum of the areas under the emission spectrum from 300–345 and from 435–480 nm, at an excitation of 254 nm.	An indicator of the extent of humification and molecular complexity.
Biological Index^11^	The ratio of emission intensity at 380 and 430 nm, at an excitation of 310 nm.	An indicator of autotrophic productivity.
Freshness Index^12^	The emission intensity at 380 nm divided by the maximum emission intensity between 420 and 435 nm, at an excitation of 310 nm.	An indicator of recently-produced material.
Peak A	Fluorescence intensity at an excitation of 260 nm and an emission of 450 nm.	Terrestrial humic-like
Peak C	Fluorescence intensity at an excitation of 340 nm and an emission of 440 nm.	Ubiquitous humic-like
Peak M	Fluorescence intensity at an excitation of 300 nm and an emission of 390 nm.	Microbial humic-like
Peak B	Fluorescence intensity at an excitation of 275 nm and an emission of 310 nm.	Protein-like; tyrosine-like
Peak T	Fluorescence intensity at an excitation of 275 nm and an emission of 340 nm.	Protein-like; tryptophan-like

## Data Records

The data records include measurements for giant miscanthus leaf and stem chemical composition and WEOM characterization. Each data record includes identifying information regarding its sample number, circular plot location, replicate number, latitude and longitude coordinates, and whether the sample was taken from leaf or stem tissues. Chemical composition measurements include the total macro- and micronutrients described above, with corresponding MDL values, nutrient concentrations with MDL values shown where true values were less than the MDL, and true concentration values, for each nutrient. Dry weight is provided for each sample, and C and N concentrations are provided as a percentage of dry weight. For stems, length is provided. Spectroscopic measurements follow for each record, including the absorbance at 254 nm (Abs254), the ratios of the absorbance at 254 and 365 nm (Abs254:365) and at 280 and 465 nm (Abs280:465), the spectral slope ratio (S_R_), the fluorescence index (FI), the humification index (HIX), the biological index (BIX), the freshness index (β:α), and the fluorescence intensity peaks A, C, M, B, and T.

Data records are archived in the USDA National Agricultural Library, Ag Data Commons repository and are publicly available^14^. Data files include identical versions of the data in two formats: a .xlsx file and a .xml file. A data dictionary.txt file provides metadata for the.xml version, while an identical metadata tab is included in the .xlsx.

The raw spectroscopic data are available as .dat files from the corresponding author upon request.

## Technical Validation

### Digestion procedures and ICP analysis

**Fig. 3:**
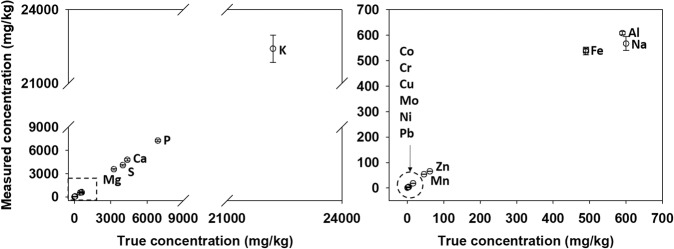
Comparison between the true and the measured nutrient concentrations of the Soybean Meal CRM. Left panel – entire concentration range for the nutrients quantified. Right panel - an expansion showing the low concentration range of the nutrients quantified.

All digestion vessels were cleaned between batches with soap and water followed by the CEM Xpress Clean method and a thorough deionized water rinse. To establish digestion proficiency and baseline performance of the microwave digestion system, a Soybean Meal Certified Reference Material (CRM-SBM-S, High Purity Standards, North Charleston, South Carolina, USA) was digested in duplicate with each batch (40 samples per batch) and the average percent recovery for all macro- and micronutrients was calculated. All nutrients were recovered with less than 10% error and the percent recoveries ranged from 95% for Al to 109% for Ca, Fe, Mg, and Mo. To assess and visualize the accuracy of the digestion and ICP analyses, a comparison was made between the true, certified nutrient concentrations of the Soybean Meal CRM (reported in the certificate of analysis) and the measured CRM nutrient concentrations (Fig. 3). The low standard deviations observed between the true and measured concentrations indicate the accuracy and precision of the digestion and ICP procedures.

All the standards used for calibration of the ICP were prepared gravimetrically and nutrient concentrations were determined in mg/kg (ppm). A calibration curve was run at the beginning of each ICP sequence with calibration ranges from 0.001 mg/kg to 100 mg/kg. Because of variable concentrations in the samples, some nutrients required low (S, Se, Si, and V) and high calibration points (B, Cd, Co, Cr, Mo, Ni, Pb, Se, Ti, V, and Zn). Samples were run with 1 and 10 ppm calibration verification standards that were added to the sequence to monitor the stability and accuracy of the calibration. Duplicate analyses of the Soybean Meal CRM were run with each instrument sequence to monitor accuracy and precision.

A blank-based MDL was calculated as the mean blank plus t times the standard deviation of all the blanks digested and analysed for this study. The greater of the blank-based MDL and the low calibration point was multiplied by the dilution factor for each sample to generate accurate, sample-specific detection limits for each analyte.

### Spectroscopic data

Although optical measurements are simple and straightforward, they can be affected by many variables such as inconsistencies in sample preparation (e.g., filter pore size, sample storage and preservation), in the correction of interferences (e.g., sample dilution, inner filter corrections), and in dealing with instrument drift (lamp intensity). The optical dataset reported here was obtained by following a documented, standardized procedure for the operation of the Aqualog spectrofluorometer to ensure that the optical measurements collected are useful and widely comparable^15^. On each day of sample analysis, the instrument was warmed up for 1 hour prior to the first scan. Lamp hours were recorded as soon as the instrument was turned on. Because the lamp degrades over time, it is routinely replaced annually regardless of hours of operation (the manufacturer recommends changing the lamp after 1000 hours of use). Gloves were always worn when handling the cuvettes and samples. All laboratory blanks and sample dilutions were prepared with deionized water (18.2 MΩ resistance, ≤ 5 ppb total organic C) produced with a Milli-Q Advantage A10 water purification system (Millipore Sigma, Burlington, Massachusetts, USA). To avoid sample contamination due to bacterial breakthrough from resin beds, activated C, and filters, the water purification system used in the lab was maintained and monitored frequently for background C and bacterial growth. The cuvette was rinsed between each sample with plenty of deionized water.

To track and maintain instrument performance, two validation experiments were run on each day of analysis: The excitation validation scan and the water Raman signal-to-noise ratio and emission calibration scan. The excitation validation scan was used to verify lamp performance and peak position at 467 ± 1 nm. The water Raman signal-to-noise and emission calibration scan examines the wavelength calibration of the CCD detector and consists in the emission scan of the Raman-scatter band of water^15^. This scan was used to verify the peak location of the water Raman at 397 ± 1 nm and the Raman peak area was used to normalize the fluorescence signals, producing data that can be compared across instruments. Multiple laboratory blanks (6–9) were analysed on each day of analysis to obtain the signal associated with the instrument, cuvette, and deionized water in the absence of fluorescent WEOM. The daily blanks were subtracted from the analytical set run on the same day.

Optical measurements of highly concentrated samples can be subject to measurement errors such as inner filter effects^7^. The inner filter effect has been found to be linear and correctable for natural water samples when the Abs254 is between 0.03–0.3 absorbance units (AU) when measured in a 1 cm cuvette^10,16^. To standardize the correction procedure used for this dataset, the WEOM samples were initially analysed at full concentration and the Abs254 was recorded. If the Abs254 exceeded 0.3 AU, the sample was diluted to a concentration at which the Abs254 was in the range of 0.3–0.3 AU and corrections for the inner filter effect were applied.

## Usage Notes

Pertaining to the macro- and micronutrient data: To ascertain which concentration records are lower than the corresponding MDL, a comparison of the XX_Conc_MDL and XX_Conc_True fields can be done, where affected records are true when XX_Conc_True is less than XX_Conc_MDL (XX is the element name).

The data are ready for use with a geographic information system (GIS), where longitude and latitude coordinates are projected as noted above. Since exact sample point locations along transects were not measured in the field, users are advised to consider the replicate values as pertaining to the area of the circular plot (r ≈ 8 m) at the single sampling location provided by the coordinates.

## Data Availability

No custom code was used for the generation or analysis of this data.
